# Exposure and Reactions to Cancer Treatment Misinformation and Advice: Survey Study

**DOI:** 10.2196/43749

**Published:** 2023-07-28

**Authors:** Allison J Lazard, Sydney Nicolla, Rhyan N Vereen, Shanetta Pendleton, Marjory Charlot, Hung-Jui Tan, Dominic DiFranzo, Marlyn Pulido, Nabarun Dasgupta

**Affiliations:** 1 Lineberger Comprehensive Cancer Center University of North Carolina at Chapel Hill Chapel Hill, NC United States; 2 Hussman School of Journalism and Media University of North Carolina at Chapel Hll Chapel Hill, NC United States; 3 Department of Medicine Division of Oncology, School of Medicine University of North Carolina at Chapel Hill Chapel Hill, NC United States; 4 Department of Urology School of Medicine University of North Carolina at Chapel Hill Chapel Hill, NC United States; 5 P.C. Rossin College of Engineering and Applied Science Lehigh University Bethlehem, PA United States; 6 Gillings School of Global Public Health University of North Carolina at Chapel Hill Chapel Hill, NC United States; 7 Injury Prevention Research Center University of North Carolina at Chapel Hill Chapel Hill, NC United States

**Keywords:** cancer, misinformation, social media, prosocial intervening, treatment, false information, alternative medicine, information spread, dissemination, infodemiology, mobile phone

## Abstract

**Background:**

Cancer treatment misinformation, or false claims about alternative cures, often spreads faster and farther than true information on social media. Cancer treatment misinformation can harm the psychosocial and physical health of individuals with cancer and their cancer care networks by causing distress and encouraging people to abandon support, potentially leading to deviations from evidence-based care. There is a pressing need to understand how cancer treatment misinformation is shared and uncover ways to reduce misinformation.

**Objective:**

We aimed to better understand exposure and reactions to cancer treatment misinformation, including the willingness of study participants to prosocially intervene and their intentions to share Instagram posts with cancer treatment misinformation.

**Methods:**

We conducted a survey on cancer treatment misinformation among US adults in December 2021. Participants reported their exposure and reactions to cancer treatment misinformation generally (saw or heard, source, type of advice, and curiosity) and specifically on social media (platform, believability). Participants were then randomly assigned to view 1 of 3 cancer treatment misinformation posts or an information post and asked to report their willingness to prosocially intervene and their intentions to share.

**Results:**

Among US adult participants (N=603; mean age 46, SD 18.83 years), including those with cancer and cancer caregivers, almost 1 in 4 (142/603, 23.5%) received advice about alternative ways to treat or cure cancer. Advice was primarily shared through family (39.4%) and friends (37.3%) for digestive (30.3%) and natural (14.1%) alternative cancer treatments, which generated curiosity among most recipients (106/142, 74.6%). More than half of participants (337/603, 55.9%) saw any cancer treatment misinformation on social media, with significantly higher exposure for those with cancer (53/109, 70.6%) than for those without cancer (89/494, 52.6%; *P*<.001). Participants saw cancer misinformation on Facebook (39.8%), YouTube (27%), Instagram (22.1%), and TikTok (14.1%), among other platforms. Participants (429/603, 71.1%) thought cancer treatment misinformation was true, at least sometimes, on social media. More than half (357/603, 59.2%) were likely to share any cancer misinformation posts shown. Many participants (412/603, 68.3%) were willing to prosocially intervene for any cancer misinformation posts, including flagging the cancer treatment misinformation posts as false (49.7%-51.4%) or reporting them to the platform (48.1%-51.4%). Among the participants, individuals with cancer and those who identified as Black or Hispanic reported greater willingness to intervene to reduce cancer misinformation but also higher intentions to share misinformation.

**Conclusions:**

Cancer treatment misinformation reaches US adults through social media, including on widely used platforms for support. Many believe that social media posts about alternative cancer treatment are true at least some of the time. The willingness of US adults, including those with cancer and members of susceptible populations, to prosocially intervene could initiate the necessary community action to reduce cancer treatment misinformation if coupled with strategies to help individuals discern false claims.

## Introduction

### Background

Cancer misinformation shared through word of mouth and on social media is harmful to individuals with cancer, as well as cancer care networks made of friends, family, and individuals who support them [1,2]. Cancer misinformation comprises claims that are not supported by current scientific consensus [2,3]. Specifically, cancer treatment misinformation includes false, exaggerated, or misleading claims about cancer treatments and cures. Individuals with cancer and their care networks receive unwanted advice through cancer misinformation directly from individuals they know and on social media [4,5]. Social media posts with cancer information have been found to contain 30% to 80% misinformation, generally, with treatment-related posts containing more misinformation than other types of cancer support [6-9].

Cancer treatment misinformation harms the psychological health of individuals with cancer and their care networks by increasing distress, self-doubt, or decisional regret [4,10]. Social support can also be disrupted if individuals feel pressured to abandon relationships and resources to avoid exposure to cancer treatment misinformation [4,5]. Cancer misinformation is also potentially harmful to physical health if one acts on treatment misinformation by deviating from evidence-based care plans or using untested supplements, diets, or therapies commonly found on social media [11-14]. Emerging evidence suggests that patients may have over a 2-fold increased risk of death if they abandon evidence-based clinical care for false cures [13,15] and that addressing misinformation for treatment decisions could increase survival by more than 5 times among some cancers [9,13,15]. Moreover, the physical and mental health of individuals with cancer is strained when people in their care networks are distressed by cancer misinformation and care burdens [10].

Cancer misinformation spreads farther and faster than accurate information on social media through public posts and private messages in the United States [6]. Most US adults own or have access to a smartphone (85%) [16], use the web daily (85%) [17], and use visual-based social media (81%) [18]. Individuals use social media to seek cancer-related information and immediate answers for themselves or to support their loved ones in treatment or survivorship [4,19-21]. After diagnosis, individuals with cancer and their care networks receive more web-based cancer misinformation at higher frequencies [4,22] and are particularly susceptible when experiencing stress and despair when cancer advances or recurs or is not responsive to the treatment. Unfortunately, many in cancer care networks amplify harmful cancer misinformation with good intentions [20,23,24]; this misguided altruism should be redirected to support community action to prosocially intervene, including removing or refuting false claims, to reduce cancer misinformation.

### Objectives

Understanding exposure and reactions to cancer misinformation is critical for developing responsive social media designs to encourage prosocial intervention, instead of sharing, to reduce misinformation. In this study, we asked US adults about cancer misinformation exposure to better understand where this information comes from and the types of unwanted advice to answer the following research questions: (1) Are people receiving advice for cancer treatment misinformation? If yes, from whom and what is the advice? (2) Are individuals who receive cancer treatment misinformation curious about these alternative treatments or cures? and (3) Are people exposed to cancer treatment misinformation on social media platforms, on what platforms, and do they believe this misinformation to be true? We then explored the reactions to cancer misinformation posts on visual-based social media. US adult participants, including those with cancer and cancer caregivers, viewed 1 of 4 posts about cancer treatments and cures adapted from Instagram and reported their willingness to intervene (intended reaction) and sharing intentions (unintended reaction) to address the remaining research questions: (4) Are individuals willing to prosocially intervene with cancer treatment misinformation? What actions would people take? and (5) Do individuals intend to share cancer treatment misinformation? What are the channels?

## Methods

### Participants

We recruited a convenience sample of US adults through the Qualtrics Online Panel platform (Qualtrics LLC) from December 7, 2021, to December 10, 2021, as part of a study on health behaviors and beliefs. To be eligible for the study, individuals had to be aged ≥18 years and live in the United States (as determined via “GeoIP Estimation” on the Qualtrics platform) at the time of completing the survey. There were no additional exclusion criteria.

### Ethics Approval, Informed Consent, and Participation

The University of North Carolina Institutional Review Board approved all study procedures (#20-2338). After accessing the survey link, the participants provided informed consent by reading the approved consent form. Participants then clicked to move forward with the survey after viewing this statement: “By continuing with the survey below, you acknowledge that you have read the information on this page and agree to be in this research study.” The participants received incentives based on the reward type and amount set by the survey vendor, Qualtrics (eg, cash and reward points). To protect the privacy and confidentiality of participants, all publicly available quantitative data were deidentified, and open-ended responses were not included in those public repositories.

### Procedure

The participants provided their consent before beginning the web-based survey. Before responding to our study questions, the participants responded to items about dietary choices, the needs of families with children diagnosed with intellectual or developmental disabilities, trust in health-related information, physical activity and sleep, and access to COVID-19–related information. Participants were then given the following prompt about the focus of our study before answering any items: “We want to ask you about advice for alternative cancer treatments or cures offered by someone outside a clinical care team. Sometimes individuals offer advice about alternative ways to treat or cure cancer (e.g., shrink tumors). You may have experienced this for yourself or for someone you know with cancer. This is different from advice to treat symptoms (e.g., manage pain)” (see Appendix A in Multimedia Appendix 1 for full survey).

After reading this prompt, the participants reported their exposure to advice for alternative treatments or cures for cancer. Participants who reported past exposure to advice were given additional items regarding (1) the source of the advice, (2) a description of what was recommended, and (3) whether they were curious about the treatment or cure.

Next, all participants reported whether they had exposure to information about alternative cancer treatments or cures on social media by selecting different platforms, as well as how often they perceived this cancer information to be true. This section began with this prompt: “For these next questions, think about any advice you have been given, information shared with others, or general posts and comments on social media.”

All participants were then randomized to view 1 of 4 Instagram posts with cancer information: 3 misinformation posts (false according to scientific consensus) or 1 information post (accurate according to scientific consensus). With the stimuli shown, participants reported their willingness to prosocially intervene (intended reaction) and intentions to share (unintended reaction), regardless of which of the 4 posts they received (misinformation or information). Finally, the participants reported their demographic information, including their personal experiences with cancer or cancer caregiving. All participants viewed the same survey with 2 exceptions: (1) participants were only asked about the source, description, and curiosity that the cancer treatment advice aroused if they selected “yes” to exposure and (2) the Instagram posts were randomized so that participants only saw 1 of the 4 possible stimuli.

### Stimuli

**Figure 1:**
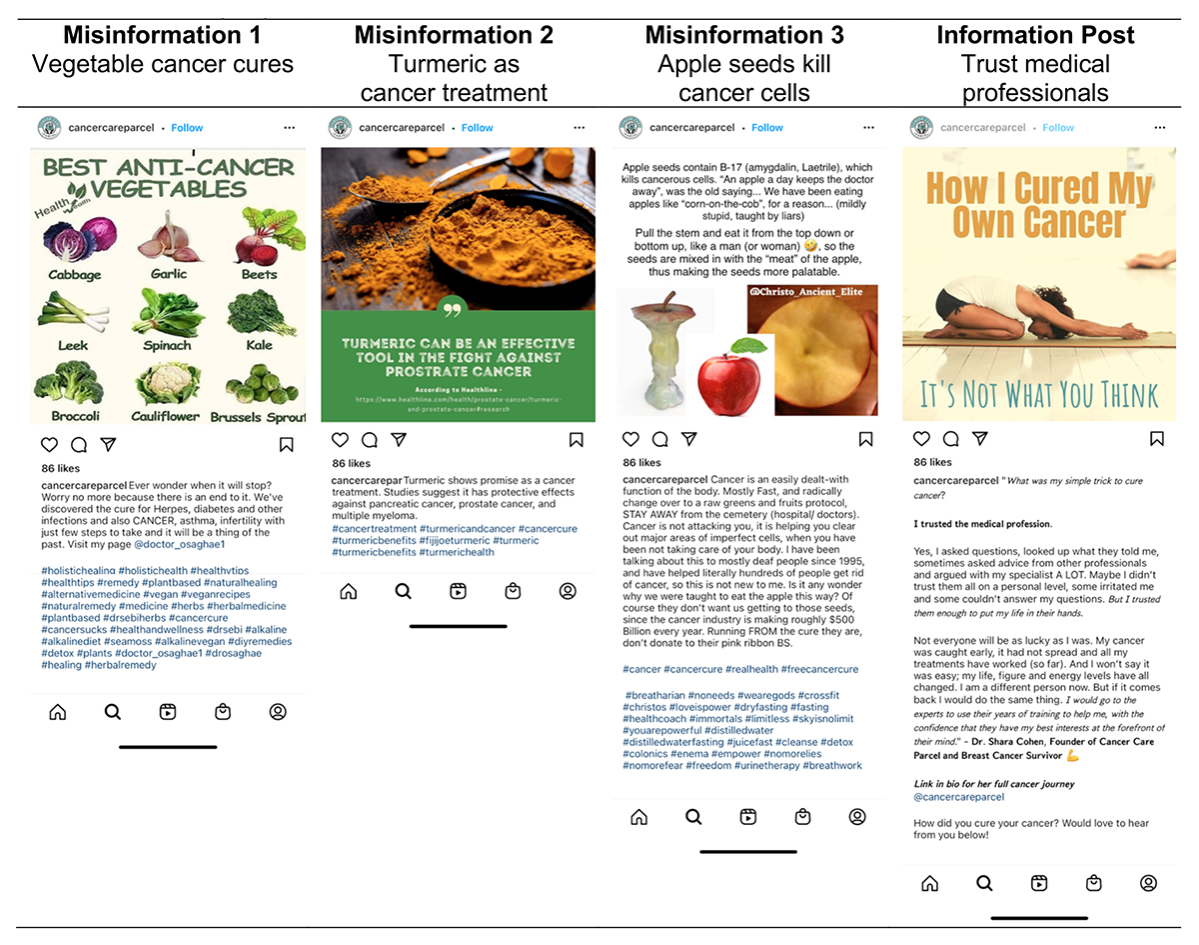
Cancer misinformation stimulus posts.

The 4 stimulus posts were adapted from cancer treatment posts found on Instagram (Figure 1). For the misinformation stimuli, we modified 3 Instagram posts that contained misinformation about false cancer treatments and cures. The original posts were all found under the hashtag #cancercure and contained highly prevalent misinformation, encouraging individuals to deviate from their current or evidence-based care by trying untested therapies or experimenting with home remedies, including recommendations for specific supplements or diets [4,6,23,25]. These misinformation stimulus posts were about vegetable cancer cures (misinformation 1), turmeric as a cancer treatment (misinformation 2), and apple seeds killing cancer cells (misinformation 3). For a comparison condition, we selected 1 Instagram post about trusting cancer medical experts for evidence-based care (information post). Screenshots of the Instagram posts were captured to retain the visuals and text as they appeared on social media; only the source and engagement metrics were updated to be consistent across stimuli (ie, the same profile photo, profile name, and number of likes).

### Measures

#### Advice for Cancer Treatment and Cures

Exposure to advice about cancer treatment was assessed with the item, “Have you seen or heard anyone offering alternative treatment or cures for cancer?” The response options were “yes,” “no,” or “not sure”; only individuals who responded “yes” were considered to have prior exposure and were asked about the following items: (1) source, (2) description of the advice, and (3) curiosity. Participants reported the source of advice with the item “Who offered advice about alternative cancer treatment or cures? Check all that apply.” The response options included “family,” friends,” “someone I know but wouldn’t consider a friend,” “someone I don’t know,” and “other, please describe.” A description of the advice was captured with the open-ended question “What was the advice for treatment of cancer cures?” Curiosity about the advice was captured with “Were you ever curious about using any alternative treatments or cures suggested for yourself or someone you know with cancer?” with the response options “never,” “sometimes,” “usually,” and “always.” Higher scores indicated greater curiosity.

#### Cancer Treatment Misinformation on Social Media

Exposure to information about alternative cancer treatments on social media was assessed with the question “Have you seen any information about alternative cancer treatments or cures on social media? Select all platforms where you have seen advice for alternative treatments and cures.” The response options included “Facebook,” “Instagram,” “Twitter,” “YouTube,” “TikTok,” “Snapchat,” “Pinterest,” “Reddit,” “other,” and “I have not seen information about alternative treatments or cures on social media.” All the participants then responded how often they perceived the information to be believable or true by replying to the question “To the best of your knowledge, how often is information about alternative cancer treatments and cures shared on social media true?” The response options were “never,” “sometimes,” “usually,” and “always.” Higher scores indicated that information was believed to be true more often.

#### Willingness to Intervene

We assessed whether the individuals would be willing to intervene to reduce cancer misinformation with 5 specific actions. Following the stem of “How likely would you be to...,” actions included the following: “flag as misinformation for others to see with system options,” “like (endorse) comments that disagree with information in this post,” “comment on the post(s) to correct untrue information,” “report as misinformation to the platform,” and “hide the untrue information so others wouldn’t see it, but the poster isn’t aware of your action (if available).” The response options were “not at all,” “a little,” “a moderate amount,” “quite a bit,” and “a great deal.” Higher scores indicated a greater willingness to prosocially intervene.

#### Sharing Intentions

We assessed whether and how people would share by asking them to follow the stem “How likely would you be to...” with “comment on the post to endorse the information,” “share with someone in a direct message,” “text it to someone,” “show someone in person,” or “post on your social media.” The response options were “not at all,” “a little,” “a moderate amount,” “quite a bit,” and “a great deal.” Higher scores indicated greater sharing intentions.

### Data Analysis

Before data collection, we preregistered this study on AsPredicted (2WN_3HD). We first analyzed descriptive results for all outcomes (eg, frequencies, means, and SDs), by cancer status (had a previous diagnosis vs no diagnosis) and assigned stimuli (1 of 3 misinformation posts or the information post), to assess the willingness to intervene and sharing intentions. For significance testing, we ran separate 2-tailed *t* tests to compare whether each Instagram misinformation post increased willingness to intervene or sharing intentions compared with the post with information about trusting evidence-based care recommendations. If there were significant differences for a misinformation post versus the information post (ie, *P*<.05), we examined individual actions to better understand how participants would intervene or share the misinformation.

We added to our preregistered analyses in 3 ways: (1) we explored whether there were differences in cancer misinformation exposure (from someone offering advice and social media), curiosity, and believability by cancer status (had a previous diagnosis vs not). We conducted chi-square tests for assessing the categorical outcomes (general exposure and social media exposure) and 2-tailed *t* tests for assessing the continuous outcomes (curiosity and believability); (2) we conducted ANOVAs to examine the main effects and moderation by cancer status (had a previous diagnosis vs not), caregiving status (was or is a caregiver vs not), race (Black vs White participants), and ethnicity (Hispanic vs non-Hispanic) on willingness to intervene or share cancer misinformation. Each subgroup was included as a predictor, along with the participants’ assigned stimuli (1 of 3 misinformation posts or the information post), in separate ANOVAs for assessing the willingness to prosocially intervene and sharing intentions; and (3) we coded the open-ended responses for types of cancer treatments and cures that the participants personally received after the data collection was complete.

Cancer treatments and cure responses were coded as “digestive,” including food, drinks, dietary supplements, or over-the-counter medication taken orally or inhaled; “natural,” including holistic, homeopathic, or natural medicine; “experiential,” including positive thinking, knowledge, mediation, yoga, or other physical activity; “location,” including traveling to a specific place; “topical,” including creams, crystals, clothing, or other items put on the body; and “clinical cancer care,” including chemotherapy, radiation, and surgery. Responses were coded for the type of treatment or cure reported (yes=1 and no=0), regardless of the direction of the advice—to use or avoid—or the nature of the advice—accurate or misinformation. Codes for types of alternative treatments and cures were not mutually exclusive except for our last code: if the treatment or cure advice was unclear (eg, lifestyle changes), this was coded as “unspecified” alternative advice only. We double coded all open-ended responses independently with 2 team members (95% agreement). When the initial codes were not in agreement, a third coder independently resolved for the majority or unanimous agreement for all codes in the final data set.

## Results

### Overview

Participants’ (N=603) average age was 46 (SD 18.83) years. See Table 1 for participant demographics and cancer characteristics and Appendix B in Multimedia Appendix 1 for demographics by stimuli exposure group. Participants identified as female (347/603, 57.5%), non-Hispanic (538/603, 89.2%), White (463/603, 76.8%), and Black or African American adults (83/603, 13.8%). Almost 1 in 5 participants (109/603, 18.1%) had a previous cancer diagnosis, and more than a third (211/603, 35%) were cancer caregivers.

**Table 1:** Participant characteristics (N=603).

Characteristics			Values
Current age (years), mean (SD)			45.74 (18.83)
**Gender^a^, n (%)**			
	Woman		347 (57.5)
	Man		247 (41.2)
	Neither woman nor man		6 (1)
**Transgender^a^, n (%)**			
	Yes, transgender		27 (4.5)
	No, not transgender		569 (95.5)
**Sexual orientation^a^, n (%)**			
	Straight or heterosexual		524 (87.6)
	Gay or lesbian		28 (4.7)
	Bisexual		46 (7.7)
**Race and ethnicity, n (%)**			
	White		463 (76.8)
	Black or African American		83 (13.8)
	American Indian or Alaska Native		12 (2)
	Asian		21 (3.5)
	Native Hawaiian or Other Pacific Islander		2 (0.3)
	Some other race		8 (1.3)
	Multiracial		14 (2.3)
**Hispanic, Latino, or Spanish ethnicity, n (%)**			
	Yes		65 (10.8)
	No		538 (89.2)
**Education, n (%)**			
	Less than high school		37 (6.1)
	High school or GED^b^		181 (30)
	Some college		162 (26.9)
	Associate’s degree		53 (8.8)
	Bachelor’s degree		107 (17.7)
	Graduate or professional degree		62 (10.3)
**Annual household income (US $)^a^, n (%)**			
	0-24,999		227 (37.8)
	25,000-49,999		168 (27.9)
	50,000-74,999		73 (12.1)
	≥75,000		134 (22.3)
**Cancer survivor, n (%)**			
	Yes		109 (18.1)
	No		494 (81.9)
	**Primary diagnosis (n=109)**		
		Bladder cancer	3 (2.8)
		Breast cancer	10 (9.2)
		Colon and rectal cancer	11 (10.1)
		Endometrial cancer	5 (4.6)
		Kidney cancer	8 (7.3)
		Leukemia	4 (3.7)
		Liver cancer	3 (2.8)
		Lung cancer	7 (6.4)
		Melanoma	8 (7.3)
		Non-Hodgkin lymphoma	4 (3.7)
		Nonmelanoma skin cancer	5 (4.6)
		Pancreatic cancer	2 (1.8)
		Prostate cancer	9 (8.2)
		Thyroid cancer	3 (2.8)
		Other cancer	22 (20.2)
**Cancer caregiver, n (%)**			
	Yes		211 (35)
	No		392 (65)
	**Relationship to the recipient of cancer care (n=211)**		
		Spouse or partner	58 (27.5)
		Parent	77 (36.5)
		Another family member	74 (35.1)
		Friend	28 (13.3)
		Other	7 (3.3)

^a^Total is <603 participants for demographic characteristics of gender (n=600), transgender people (n=596), sexual orientation (n=598), and annual household income (n=602) because of participants preferring not to report or missing data.

^b^GED: General Educational Development.

### Exposure to Misinformation for Cancer Treatments and Cures

When asked about past exposure to advice for alternative cancer treatments and cures generally, about 1 in 4 participants (142/603, 23.5%) reported receiving advice. Exposure to advice about alternative treatments and cures (ie, cancer treatment misinformation) was significantly higher among individuals with a cancer diagnosis (53/109, 48.6%) than those without (89/494, 18%) a cancer diagnosis; *c*^2^_2_=46.5, *P*<.001.

Among those exposed to misinformation (n=142), the advice for alternative treatment and cures was primarily from family (39.4%), friends (37.3%), people they did not know (27.5%), and acquaintances (21.1%). In addition, among those exposed to advice, 3 out of 4 individuals (106/142, 74.6%) were curious about these alternative cancer treatments and cures, ranging from being sometimes (43.7%) to usually (18.3%) to always (12.7%) curious. Curiosity did not differ by cancer status (t_140_=.05; *P*=.96).

Shared advice for cancer treatment and cures ranged from general to specific advice. Most advice shared was about digestive or dietary treatments (30.3%). Dietary advice included to have a “good diet,” “eat more fruits,” “vitamins,” and use “cannabis” in many forms. Dietary advice also included more problematic and potentially harmful misinformation, which included taking “non-sanctioned,” “medication,” and “dietary supplements” without US Food and Drug Administration approval; “medication that’s meant to treat dogs”; diets with “no solid foods”; and diets to “change the pH of the body.” Natural treatments and cures (14.1%), often including recommendations for herbal remedies, were the next most common alternative options. One in 10 participants (14/142, 9.9%) reported receiving some advice for clinical care, including to receive (or not receive) chemotherapy, radiation, or surgery; notably, without patient information, it is impossible to determine whether this advice follows or deviates from scientific consensus for evidence-based care. Fewer participants shared that they received experiential advice for prayer or positive thinking (9.2%), to go to a specific location like “Mexico for treatment” (4.9%), or the use of essential oils as a potential topical treatment (1.4%). About 1 in 10 participants (13/142, 9.2%) did not specify the type of treatment or cure suggested.

Participants reported higher exposure to misinformation on social media; more than half of all participants (55.9%) reported exposure to advice, information shared with others, and general posts or comments about alternative cancer treatment or cures on social media. Exposure to cancer misinformation on social media was significantly higher among those with a cancer diagnosis (70.6%) compared with those without a cancer diagnosis (52.6%; *c*^2^_8_=23.0, *P*=.003). Exposure differed by platform, with the greatest exposure on Facebook (39.8%), followed by YouTube (27%), Instagram (22.1%), TikTok (14.1%), Twitter (11.6%), Snapchat (11.6%), Pinterest (6%), and Reddit (3.3%). Although more than a quarter of the participants (28.7%) said this information was “never” true, most thought information on social media about alternative treatments and cures was sometimes (51.4%), usually (14.4%), or always (5.3%) true. Notably, individuals with cancer (mean score 2.17, SD 0.94) believed cancer treatment misinformation on social media to be true more often compared with those without a diagnosis (mean score 1.92, SD 0.76; t_141_=2.56; *P*=.01).

### Willingness to Intervene With Cancer Misinformation on Instagram

Participants were, on average, moderately willing to intervene with any action across the Instagram posts (mean score 2.35, SD 1.08; a=.861). Participants were more likely to intervene (overall) with the misinformation post about vegetable cancer cures when compared with the information post for trusting cancer medical experts (t_303_=2.03; *P*=.04; Table 2). For specific actions for the vegetable cancer cures misinformation post (vs information post), participants were more willing to flag it as misinformation (t_302_=2.11; *P*=.04) and endorse (ie, like) comments that disagreed with the post (t_303_=2.55; *P*=.01). There were no differences in the willingness to comment to correct untrue information (*P*=.88), hide the post so that others would not see (*P*=.07), or report the post as misinformation to the platform (*P*=.11). The act of intervening did not differ for the other 2 misinformation posts (vs information post) about turmeric as a cancer treatment (*P*=.32) and apple seeds killing cancer cells (*P*=.31).

**Table 2:** Willingness to intervene and sharing intentions.^a^

			All posts (N=603)				Misinformation 1, vegetables (n=148)				Misinformation 2, turmeric (n=156)					Misinformation 3, apple seeds (n=143)					Information post (n=156)		
			Score, mean (SD)		% likely		Score, mean (SD)		% likely		Score, mean (SD)		% likely		Score, mean (SD)			% likely		Score, mean (SD)			% likely
**Willingness to intervene**																							
	Overall	2.35 (1.08)		68.3		*2.48 (1.11)^b^*		70.9		2.35 (1.10)		69.9		2.35 (1.06)			69		*2.22 (1.04)*			64.1	
	Flag as misinformation	2.42 (1.37)		48.3		*2.55 (1.37)*		51.4		2.40 (1.33)		50.0		2.51 (1.45)			50		*2.22 (1.31)*			42.3	
	Endorse (like) rebuttals	2.29 (1.31)		44.3		*2.54 (1.35)*		53.4		2.36 (1.37)		44.2		2.10 (1.21)			41		*2.15 (1.27)*			39.1	
	Comment to correct	2.33 (1.33)		45.3		2.39 (1.33)		48.6		2.30 (1.34)		44.2		2.24 (1.29)			43		2.37 (1.36)			45.5	
	Report to platform	2.43 (1.37)		48.6		2.53 (1.33)		51.4		2.37 (1.34)		48.1		2.56 (1.48)			51		2.28 (1.32)			44.2	
	Hide post	2.27 (1.35)		42.3		2.37 (1.40)		44.6		2.29 (1.33)		43.6		2.35 (1.42)			45		2.10 (1.26)			35.9	
**Sharing intentions**																							
	Overall	2.31 (1.23)		59.2		2.50 (1.26)		63.5		2.36 (1.26)		60.9		2.07 (1.15)			51.7		2.29 (1.20)			56.4	
	Comment to endorse	2.25 (1.31)		43.9		2.40 (1.31)		49.3		2.31 (1.35)		45.5		2.07 (1.27)			39.9		2.22 (1.31)			41	
	Share in a direct message	2.34 (1.37)		45.6		2.55 (1.42)		53.4		2.41 (1.43)		48.1		2.08 (1.26)			35.7		2.31 (1.32)			44.9	
	Text to someone	2.32 (1.37)		44.4		2.54 (1.42)		51.4		2.31 (1.38)		44.9		2.05 (1.26)			35		2.37 (1.36)			46.2	
	Show in person	2.38 (1.36)		47.6		2.58 (1.40)		52.7		2.44 (1.39)		51.9		2.15 (1.34)			39.9		2.33 (1.30)			45.5	
	Post on social media	2.25 (1.37)		43.1		2.45 (1.42)		50		2.31 (1.37)		43.6		2.01 (1.32)			37.8		2.21 (1.35)			41	

^a^Percentage of individuals who reported they were “a moderate amount” (3) to “a great deal” (5) likely to intervene or share on a 1 to 5 scale; participants who selected “not at all” (1) or “a little bit” (2) were excluded from the percentage share. Overall, % likely represents the percentage of participants who were “a moderate amount” to “a great deal” likely to intervene or share via one or more specific actions.

^b^Italicized values indicate that they share a superscript difference by *P*<.05.

Many participants (412/603, 68.3%) reported that they were willing to intervene with the Instagram cancer misinformation posts. Specific to the 3 misinformation posts, participants were willing to “a moderate amount” to “a great deal” (3-5 on a 5-point scale) to intervene by flagging the posts as misinformation for others to see (49.7%-51.4%) and reporting the posts as misinformation to the platform (48.1%-51.4%), followed by liking a comment that disagrees with the post (40.6%-53.4%), commenting to correct untrue information (42.7%-48.6%), and hiding the post from others (43.6%-45.5%).

Being a cancer survivor or a cancer caregiver, as well as race and ethnicity, did not moderate willingness to intervene with the cancer Instagram posts (misinformation vs information). However, there was a main effect of willingness to intervene among cancer survivors; individuals with a cancer diagnosis were significantly more likely to intervene (mean score 2.57, SD 1.08) across any misinformation posts compared with those without diagnoses (mean score 2.30, SD 1.07; *F*_1595_=5.12; *P*=.02). There were also main effects of race and ethnicity. Black participants were significantly more willing (mean score 2.81, SD .92) to prosocially intervene across any misinformation posts compared with White participants (mean 2.24, SD 1.08; *F*_1539_=19; *P*<.001). Hispanic participants were more willing to intervene (mean score 2.64, SD 1.17) than non-Hispanic participants (mean score 2.31, SD 1.06; *F*_1596_=4.01; *P*=.05). Being a caregiver (vs not) did not influence the willingness to intervene overall.

### Sharing Cancer Misinformation on Instagram

Participants were, on average, had moderate sharing intentions with any Instagram posts (mean score 2.31, SD 1.23; a=.944). Sharing intentions did not differ across cancer misinformation posts. Participants reported that they would similarly share the information post on trusting cancer medical experts compared with vegetable cancer cures (*P*=.12), turmeric as a cancer treatment (*P*=.63), and apple seeds killing cancer cells (*P*=.12).

More than half of the participants (357/603, 59.2%) reported that they were willing to share the Instagram cancer misinformation posts. Specific to the 3 misinformation posts, participants were most willing to share by showing them to someone in person (39.9%-52.7%) and sending a private, direct message (35.7%-53.4%), followed by sending a text message (35%-51.4%), posting or reposting on social media (37.8%-50%), and commenting on the post to endorse the information (39.9%-49.3%).

The cancer survivor status, cancer caregiver status, race, or ethnicity did not moderate the sharing of cancer posts on Instagram (misinformation vs information). However, there was a main effect among cancer survivors; individuals with a cancer diagnosis were significantly more likely (mean score 2.58, SD 1.28) to share any misinformation posts compared with participants without diagnoses (mean score 3.0, SD 1.21; *F*_1595_=6.01; *P*=.02). Again, there were also main effects of race and ethnicity on sharing. Black participants were significantly more likely (mean score 3.05, SD 1.14) to share any misinformation posts than White participants (mean score 2.15, SD 1.20; *F*_1539_=37; *P*<.001). Hispanic participants were significantly more likely (mean score 2.78, SD 1.24) to share any misinformation posts than non-Hispanic participants (mean score 2.25, SD 1.21; *F*_1596_=10; *P*=.002). Being a caregiver (vs not) did not influence sharing intentions.

## Discussion

### Principal Findings

Cancer misinformation is shared widely in the United States, especially on social media, where false or misleading claims spread farther and faster than true information. Cancer misinformation is especially problematic when it is about alternative treatments or cures that are not supported by the current scientific consensus and are harmful [3,9]. When people turn to the internet after being diagnosed or when caring for someone, they hope to find information and support [21]. Yet many are exposed to viral, novel, shocking, and personal stories that claim to be true but are not [26]. A good proportion of cancer misinformation (77% in one study) can actually harm individuals with cancer, and too-good-to-be-true treatments and cures can impede treatment decision-making [9,13].

We found that 1 in 2 participants with cancer recalled someone offering them cancer misinformation as advice, while among all participants, about 1 in 4 witnessed or received advice for alternative cancer treatments and cures (in general, not social media specific). The misinformation about cancer treatment was often received from family and friends. Advice on dietary or natural alternative treatments was the most common. Although some pieces of advice may not harm patients unless used in lieu of conventional treatment (eg, following a healthy diet), other pieces of advice reported by participants includes potentially harmful cancer misinformation, including the use of nonsanctioned medicine, treatments developed for animals, or other supplements that are not US Food and Drug Administration approved. Advice about clinical care is a large and potentially problematic issue [1]. Any advice for substandard care could cause harm, and interest in this type of cancer misinformation may be higher among individuals with advanced cancer or individuals seeking advice not received during clinical encounters or novel treatments (eg, data for focal therapy for prostate cancer is weak but patients may want this to be a viable option for them) [27]. Notably, 3 out of 4 participants who received advice were curious about the alternative treatment or cure, indicating high interest among the participants when advice was given.

Exposure to any information or cancer treatment misinformation was more common on social media where more than half of the participants—regardless of cancer or caregiving status—recalled seeing information about alternative cancer treatments and cures. Cancer misinformation on social media platforms mirrored use patterns in the United States; exposure was the greatest on platforms used more by adults, including the most popular sites, YouTube and Facebook, followed by Instagram and other social media platforms [18]. It is not surprising that people see cancer misinformation on platforms that they see often. Our findings support calls for a stronger focus on visual-based social media sharing of cancer misinformation [28].

Problematically, misinformation for alternative cancer treatments and cures on social media is believable; more than two-thirds of the participants thought that these alternative treatments and cures were true at least some of the time. When cancer treatment misinformation is inconsistent with clinical consensus, it puts additional strain on patients and their care networks [9]; these individuals must verify the accuracy and relevance of information with their physicians and clinical care team and must verify other (potentially questionable) information on the internet. As we work to reduce cancer treatment misinformation, strategies that leverage social correction (a form of prosocial intervention) are likely to be more effective if supported with accompanying facts or sources to increase credibility and believability [29].

Stopping the spread of cancer misinformation through prosocial intervention may help reduce the harmful impact of false or misleading treatment claims, but only if people are able to discern false claims. Our findings point to an opportunity, along with a need, to encourage individuals to engage in bystander intervention with cancer misinformation. Although 2 in 3 participants, including those with cancer and caregivers, were willing to prosocially intervene with a variety of digital actions, many appeared to be poor at discerning what is true or trustworthy cancer treatment information on social media. In only one instance did the participants have higher willingness to intervene with false information than they did with true information post, with a recommendation to trust medical professionals. Furthermore, similar sharing intentions for true and false claims suggest that people need more guidance to assess the accuracy of social media posts. Because individuals skim social media posts, they often do not fully vet the accuracy of the content [30]. Thus, in our study, it is possible that the caption for the information post (ie, “How I cured my own cancer. It's not what you think.”) could be perceived as misinformation or people simply may find it difficult to discern trustworthy information.

Notably, individuals with cancer, Black participants, and Hispanic participants were generally more willing to prosocially intervene with and share all cancer posts, not only misinformation. Our findings suggest a greater engagement with cancer misinformation on social media among those directly affected by cancer and racially and ethnically diverse populations. Emerging evidence suggests that Black and Hispanic individuals have more exposure to health misinformation than White individuals [31]. In this context, our findings support that populations who are more susceptible are also more likely to initiate community action and sharing. Individuals who identify as Black or Hispanic or have cancer are likely to have unique motivations for using social media and intervening with misinformation. It is possible that more exposure motivates action to protect one’s community, especially in minoritized populations with health disparities [32]. In other words, Black and Hispanic individuals may be more willing to not only intervene but also share (to alert or support people in their community) health misinformation when their needs are not met by others in power [31,33]. These groups may also be more open to using social media to compensate for poor patient-provider communication or because of medical mistrust—disproportionately experienced by racial and ethnic minoritized populations—and subsequently, are more likely to intervene when informed about false information to counteract or respond to past negative experiences with or perceptions of the medical system [34,35]. Future intervention efforts to reduce cancer misinformation should be tailored and culturally relevant for these individuals, who are most likely to be affected by and are willing to address cancer misinformation on social media.

Prosocial intervention could reduce harmful cancer treatment misinformation from reaching a susceptible audience and quell the overflow of digital cancer-related content to allow for good and helpful information to reach those in need if individuals are better able to identify false claims that warrant action. Social media can be an instrumental resource for finding answers to cancer-related questions, and people can witness others who share their cancer experience and find peer support potentially unavailable with in-person networks [5,36]. Prosocial intervention would likely be most effective if used alongside other strategies, such as low-cost prompts to help people discern false claims and cancer advocacy groups providing true, reliable content on social media or myth-busting accounts (eg, #CancerRealTalk organized by cancer clinicians, patients, and advocates) across platforms [37]. Improving a combination of community efforts to reduce misinformation and encourage helpful support on social media is critical for the health and wellness of individuals with cancer and their care networks.

Individuals are more willing to intervene through simple actions. If the options are available on social media, participants were most often willing to flag posts as misinformation or report to the platform to signal inaccurate claims. Although social media users can refute claims by commenting or supporting (liking) others’ rebuttals [38], our findings indicate that individuals may be less likely to take these direct, and potentially confrontational, actions. People were somewhat less likely to like comments that refuted misinformation or comment to correct untrue claims. Thus, reducing cancer misinformation through unique, indirect platform affordances, such as flagging and reporting, appears to be more promising. These prosocial interventions have been part of effective digital bystander interventions, with increasing evidence of their ability to encourage supportive community action in the face of misinformation that perpetuates injustice, harassment, and harm [39-43]. However, individuals must be able to discern what is misinformation and know how to act; we need prompts and messages to help people question suspicious information and direct community action, as knowing how to intervene is a critical step in the human-computer interaction applications of the bystander intervention model [41]. Thus, we should consider using both prompts to serve as cues to critically assess accuracy (or at least pause to question whether the information is true)—a strategy shown to reduce sharing of false information on social media and misperceptions—and messages to counter misinformation with accurate facts to reduce misperceptions [38,44,45].

The spread of cancer misinformation is amplified by sharing on social media and offline. Sharing about a health issue or behavior interpersonally is associated with people taking the recommended actions highlighted in the message [46]. Unfortunately, more than half of our participants intended to share cancer treatment misinformation posts, causing concern about future engagement with unevidenced behaviors. Most people reported that they would share through untraceable or offline channels, as we have found with cancer prevention messages for adolescents, where most would share in person, via text message, or in ephemeral postings [47]. This could indicate that participants want to share in discreet or less-public ways. People may want to share privately to protect their image or explore curiosities without public scrutiny (eg, someone with clinician’s recommendations for radiation for prostate cancer might want to investigate a cancer treatment misinformation post but not want that to be widely known). Furthermore, these sharing methods do not leave behind trace data that could be investigated using social media data mining and analyses. To assess the implications of cancer misinformation sharing, we need multivariable approaches to ensure valid measures that account for both digital and offline sharing behaviors.

Sharing cancer misinformation may not always be intentional. In general, people share because they believe that they possess information (usually novel information) that can benefit others in their social network (eg, altruism) and not because they want to cause harm [48,49]. This may have been the case among the participants in our study. Although we did not ask for the motivation for sharing the messages in this study, the fact that a relatively high proportion of participants were willing to intervene when exposed to misinformation makes it possible that those who would share the information believed that they were positively impacting their community. Future research should address motivations to share (eg, endorse vs counter), along with sharing intentions, to better understand how misinformation is being shared in cancer networks.

### Limitations

This study has limitations. Our study is limited to the responses among a convenience sample of US adults; other populations likely have different rates of exposure to cancer misinformation and may have different reactions to social media posts. Without recruiting specifically for individuals with cancer experience (diagnosis or caregiving), we had many individuals with cancer experience in this study. It is possible that we had more individuals with cancer because of the age of the participants. One-third of the participants (34%) were aged ≥55 years, the age group that accounts for 82% of new cancers in the United States [50]. Participants self-reported caregiving by whether they had “ever cared for someone with cancer” in our survey, which may have been interpreted broadly as contributing to any level of care by some participants. Thus, we do not know whether the individuals were the primary caregivers or part of a cancer care network. In this study, receiving cancer treatment misinformation as advice (general exposure) was reported by fewer individuals than exposure to the same on social media; however, these findings are limited by the wording used in our survey. We asked about general exposure as “anyone offering advice” (potentially interpreted as only direct advice), whereas the social media exposure item was “any information” seen by participants (potentially interpreted as including both passive information and direct advice). More research is needed to determine the best way to ask about cancer misinformation exposure without biasing participants. We asked about our stimulus Instagram posts specifically; prosocial interventions and sharing intentions may differ with other messages or on other social media platforms. We also asked about the willingness for prosocial intervention and sharing intentions but did not assess behavior. In addition, we asked about intervening and sharing a unidirectional item (ie, response options ranged from no action—not at all likely—to increasing likelihood of action), which may have influenced our findings. Future research with a neutral midpoint in the response options should be explored. Future studies should also assess actual, rather than expected, participant intervention with and sharing of cancer misinformation to better understand reactions. Participants were not debriefed in this study; information to help the participants discern the validity of stimuli after participation will be incorporated into future studies. Finally, without patient information, we do not know if all clinical care recommendations align with recommended care (eg, gene therapy is clinical care but may not be recommended if there is no evidence of benefit with particular diagnoses). Additional participant information would be needed to determine whether the clinical advice is misinformation.

### Conclusions

In conclusion, cancer treatment misinformation exposure on social media is high in the United States, including visual-based social media and platforms that are widely used for cancer support. We found that many people believe cancer treatment posts on social media to be true at least some of the time, making them susceptible to potential psychosocial or physical harms of false cancer treatments and cures. In this study, 2 in 3 US adults were willing to prosocially intervene with any cancer treatment misinformation, but almost as many were also willing to share this misinformation, and few discerned between false and true claims. With strategies to encourage individuals to identify and prioritize intervening with harmful misinformation posts, there is potential to encourage community action to reduce exposure and negative impact. Susceptible populations—individuals with cancer, Black individuals, and Hispanic individuals—warrant special attention, as they are both more willing to not only prosocially intervene (intended outcome) but also share (unintended outcomes) cancer treatment misinformation.

## Appendix

Multimedia Appendix 1 Appendix A: Survey items; Appendix B: Participant characteristics by stimuli exposure group (N=603).

## Data Availability

The data sets generated during and analyzed during this study are available in the Carolina Digital Repository [51].
