# Supplementary material for: Exposure and Reactions to Cancer Treatment Misinformation and Advice: Survey Study
Source: JMIR Cancer. 2023 Jul 28;9:e43749. doi: 10.2196/43749 (PMC10422174; doi:10.2196/43749)
Supplement: Multimedia Appendix 1 [file cancer_v9i1e43749_app1.pdf]

Exposure and reactions to cancer treatment (mis)information and advice: Online survey
Lazard, Nicolla, Vereen, Pendleton, Charlot, Tan, DiFranzo, Pulido, & Dasgupta

Appendix A. Survey items

Construct	Item	Response options
Prompt	**[Page break]**<br>In this section, we want to ask you about advice for alternative cancer treatments or cures offered by someone outside a clinical care team.<br><br>Sometimes individuals offer advice about alternative ways to treat or cure cancer (e.g., shrink tumors). You may have experienced this for yourself or for someone you know with cancer. This is different from advice to treat symptoms (e.g., manage pain).	
Exposure (general)	Have you seen or heard anyone offering advice for alternative treatments or cures for cancer?	1 = Yes<br>2 = No<br>3 = Not sure
Source	**[Item only shown if "yes" is selected to Exposure]**<br>Who offered advice about alternative treatments or cancer cures? Check all that apply.	1 = Family<br>2 = Friends<br>3 = Someone I know but wouldn't consider a friend<br>4 = Someone I don't know<br>5 = Other, please describe **[allow text entry]**
Description	**[Item only shown if "yes" is selected to Exposure]**<br>What was the advice for treatment or cancer cures? Add a description of any advice below.	**[Open-ended response]**
Curiosity	**[Item only shown if "yes" is selected to Exposure]**<br>Were you ever curious about using any alternative treatments or cures suggested for yourself or someone you know with cancer?	1= Never<br>2= Sometimes<br>3= Usually<br>4= Always
Prompt	**[Page break]**<br>For these next questions, think about any advice you have been given, information shared with others, or general posts and comments on social media.	

Exposure (social media)	Have you seen any information about alternative cancer treatments or cures on social media? Select all platforms where you have seen advice for alternative treatments and cures.	1= Facebook<br>2= Instagram<br>3 = Twitter<br>4 = YouTube<br>5 = TikTok<br>6 = Snapchat<br>7 = Pinterest<br>8 = Reddit<br>9 = Other **[allow text entry]**<br>10 = I have not seen information about alternative treatments or cures on social media
Believability	To the best of your knowledge, how often is information about alternative cancer treatments and cures shared on social media true?	1= Never<br>2= Sometimes<br>3= Usually<br>4= Always
Prompt	**[Page break. Participants assigned to view one of the four Instagram posts with the Willingness to prosocially intervene and Sharing intentions items shown below, in a random order.]**<br>If you were to see this post on social media, how likely would you be to…	
Willingness to prosocially intervene	flag it as misinformation for others to see with system options (if available)?	1 = Not at all<br>2 = A little bit<br>3 = A moderate amount<br>4 = Quite a bit<br>5 = A great deal
	like (endorse) comments that disagree with information in<br>this post?	
	comment on the post to correct untrue information?	
	report as misinformation to the platform?	
	"hide" the post so other users wouldn't see but the poster isn't aware of your action (if available)?	
Sharing intentions	comment on the post to endorse the information?	1 = Not at all<br>2 = A little bit<br>3 = A moderate amount<br>4 = Quite a bit<br>5 = A great deal
	share with someone in a direct message?	
	text it to someone?	
	show someone in person?	
	post on your social media?	

Note: Programming notes are in bold brackets.

Appendix B. Participant characteristics by stimuli exposure group (*N*=603)

	**Misinformation 1, Vegetables** *n* = 148		**Misinformation 2, Turmeric** *n* = 156		**Misinformation 3, Apple seeds** *n* = 143		**Information Post** *n* = 156	
	n	%	*n*	%	*n*	%	*n*	%
Current age *M* (*SD*)	46.18	(18.83)	44.80	(18.87)	46.50	(20.14)	45.56	(17.63)
Gender								
Woman	88	59%	98	63%	78	55%	83	53%
Man	58	39%	57	37%	62	43%	70	45%
Neither woman nor man	1	1%	1	1%	2	1%	3	2%
Transgender								
Yes, transgender	7	5%	7	4%	5	3%	8	5%
No, not transgender	141	95%	149	96%	133	93%	146	94%
Sexual orientation								
Straight or heterosexual	130	88%	139	89%	116	81%	139	89%
Gay or lesbian	6	4%	8	5%	8	6%	6	4%
Bisexual	11	7%	7	4%	19	13%	9	6%
Race and Ethnicity								
White	118	80%	111	71%	107	75%	127	81%
Black or African American	20	14%	29	19%	16	11%	18	12%
American Indian or Alaska Native	2	1%	5	3%	1	1%	4	3%
Asian	2	1%	4	3%	12	8%	3	2%
Native Hawaiian or Other Pacific Islander	0	--	1	1%	0	--	1	1%
Some other race	3	2%	1	1%	3	2%	1	1%
Multiracial	3	2%	5	3%	4	3%	2	1%
Hispanic, Latino, or Spanish ethnicity								
Yes	17	11%	19	12%	12	8%	17	11%
No	131	89%	137	88%	131	92%	139	89%
Household Income								
$0-$24,999	56	38%	66	42%	44	31%	61	39%
$25,000-$49,999	42	28%	52	33%	44	31%	40	26%
$50,000-$74,999	17	11%	11	7%	27	19%	18	12%
$75,000+	43	29%	27	17%	27	19%	37	24%
Education								
Less than high school	9	6%	9	6%	6	4%	13	8%

High school or GED	39	26%	58	37%	39	27%	45	29%
Some college	33	22%	44	28%	45	31%	40	26%
Associate's degree	19	13%	11	7%	11	8%	12	8%
Bachelor's degree	28	19%	21	13%	25	17%	33	21%
Graduate or professional degree	20	14%	13	8%	16	11%	13	8%
Cancer survivor								
Yes	35	24%	31	20%	24	17%	19	12%
No	113	76%	125	80%	119	83%	137	88%
Cancer caregiver								
Yes	53	36%	56	36%	47	33%	55	35%
No	92	62%	97	62%	94	66%	97	62%

Note: Totals fewer than 603 participants for demographic characteristics of gender, transgender, sexual orientation, and annual household income are due participants preferring not to report or missing data.
